# Supplementary material for: Splice-Junction-Based Mapping of Alternative Isoforms in the Human Proteome
Source: Cell Rep. Author manuscript; Available in PMC 2020 Jan 15. (PMC6961840; doi:10.1016/j.celrep.2019.11.026)
Supplement: 2 [file NIHMS1546469-supplement-2.zip › DF1/PXD010154/Testis/Testis_17_SMARCC1_PGDVQGPGTAVGATGSGIAAAAAGLAVYR.pdf]

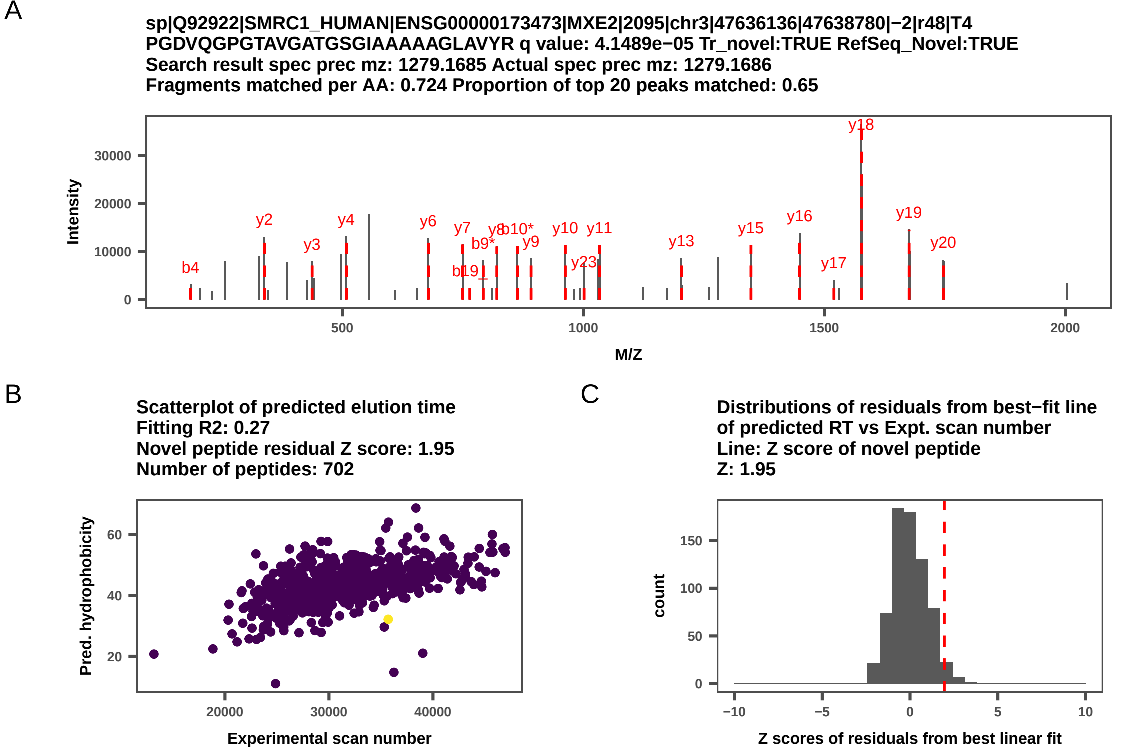
A
sp|Q92922|SMRC1_HUMAN|ENSG00000173473|MXE2|2095|chr3|47636136|47638780|−2|r48|T4
PGDVQGPGTAVGATGSGIAAAAAGLAVYR q value: 4.1489e−05 Tr_novel:TRUE RefSeq_Novel:TRUE
Search result spec prec mz: 1279.1685 Actual spec prec mz: 1279.1686
Fragments matched per AA: 0.724 Proportion of top 20 peaks matched: 0.65
Intensity
0
10000
20000
30000
b4
y2
y3
y4
y6
y7
b19
b9*
y8
b10*
y9
y10
y23
y11
y13
y15
y16
y17
y18
y19
y20
500
1000
1500
2000
M/Z
B
Scatterplot of predicted elution time
Fitting R2: 0.27
Novel peptide residual Z score: 1.95
Number of peptides: 702
Pred. hydrophobicity
20
40
60
20000
30000
40000
Experimental scan number
C
Distributions of residuals from best−fit line
of predicted RT vs Expt. scan number
Line: Z score of novel peptide
Z: 1.95
count
0
50
100
150
−10
−5
0
5
10
Z scores of residuals from best linear fit
